# Disambiguity and Alignment: An Effective Multi-Modal Alignment Method for Cross-Modal Recipe Retrieval

**DOI:** 10.3390/foods13111628

**Published:** 2024-05-23

**Authors:** Zhuoyang Zou, Xinghui Zhu, Qinying Zhu, Hongyan Zhang, Lei Zhu

**Affiliations:** College of Information and Intelligence, Hunan Agricultural University, Changsha 410128, China; zzy@stu.hunau.edu.cn (Z.Z.); zhuxh@hunau.edu.cn (X.Z.); zhuqy@stu.hunau.edu.cn (Q.Z.); hongyan_zhang@hunau.edu.cn (H.Z.)

**Keywords:** cross-modal recipe retrieval, multi-modal alignment, food image ambiguity, deep learning

## Abstract

As a prominent topic in food computing, cross-modal recipe retrieval has garnered substantial attention. However, the semantic alignment across food images and recipes cannot be further enhanced due to the lack of intra-modal alignment in existing solutions. Additionally, a critical issue named food image ambiguity is overlooked, which disrupts the convergence of models. To these ends, we propose a novel Multi-Modal Alignment Method for Cross-Modal Recipe Retrieval (MMACMR). To consider inter-modal and intra-modal alignment together, this method measures the ambiguous food image similarity under the guidance of their corresponding recipes. Additionally, we enhance recipe semantic representation learning by involving a cross-attention module between ingredients and instructions, which is effective in supporting food image similarity measurement. We conduct experiments on the challenging public dataset Recipe1M; as a result, our method outperforms several state-of-the-art methods in commonly used evaluation criteria.

## 1. Introduction

With rising awareness of health and sustainability, issues such as food safety [1,2] and nutrition [3] have gained unprecedented attention. Food computing [4,5,6,7,8] plays a crucial role in promoting healthier lifestyles, mitigating food waste, and enhancing both the quality and safety of food products. Cross-modal recipe retrieval [9,10] is one of the hot topics in food computing, leveraging artificial intelligence (AI) [11,12] which aims to retrieve the corresponding recipes by queries of food images or vice versa. In this task, food images depict finished dishes, while recipes comprise text encompassing three key components: a title, a list of ingredients, and detailed instructions outlining the cooking process.

The principal challenge in cross-modal recipe retrieval lies in mitigating the inherent heterogeneity between two distinct modalities: the recipes and the food images. To solve this challenging task, numerous studies have delved into additional interactions between the two modalities. For instance, Refs. [13,14,15] tried to learn the consistent feature distribution of food images and recipe texts. Refs. [16,17,18,19] boosted the interaction between two modalities through cross-modal attention. Ref. [20] employed a joint transformer encoder to promote alignment. Due to the complexity of image–recipe pairs, many existing studies focused on exploiting the latent semantic information within a modality. As typical studies, refs. [21,22,23,24,25] aimed to focus on the crucial term within recipes, while others [26,27,28] attempted to capture the salient objects or regions from food images to improve the cross-modal similarity measurement. Due to the complexity of the textual structure in recipes, other researchers [29,30,31,32,33] investigated the interaction among the title, ingredients, and instructions to excavate important semantics. Furthermore, some studies introduced diverse augmentation mechanisms to enhance cross-modal feature representations. For example, Refs. [34,35,36,37,38] employed various Generative Adversarial Networks (GANs) to reconstruct information from food images and recipes to bridge the heterogeneity gap across modalities, while refs. [39,40] leveraged multilingual translation to enrich the recipe information. Crowdsourcing strategy is also used to construct program representations of recipes [41]. Thanks to the flourishing development of visual language pre-training recently, some pioneers [42,43,44,45] have further embedded complex semantic relationship information into common feature subspace by leveraging the pre-trained Contrastive Language–Image Pre-Training model (CLIP).

**Figure 1:**
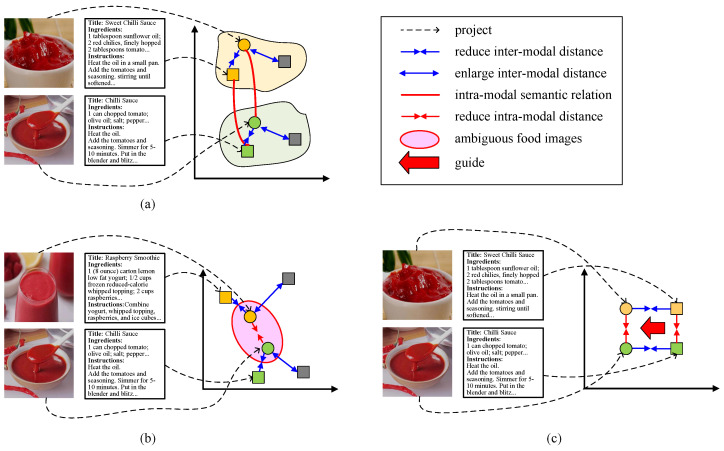
The demonstration of multi-modal alignment schemes for cross-modal recipe retrieval. (**a**) The prevailing learning strategy that ignores intra-modal alignment. (**b**) The food image ambiguity issue. (**c**) Our solution (negative samples are omitted). Circles represent images, and squares represent recipes. Shapes of the same color indicate positive pairs, while gray shapes indicate negative samples.

Despite the significant progress made so far, there is still room for further improvement in semantic distribution alignment across food images and recipes. To be specific, the prevailing efforts [18,29,30] concentrate on exploring inter-modal semantic alignment using conventional metric learning strategies, such as triplet loss. As shown in Figure 1a, the conventional metric learning strategy is devoted to reducing the distance between positive image–recipe pairs (the circles and squares with the same color) and enlarging the distance between negative samples (the circles and the gray squares) and is proficient in learning similarity relations within each image–recipe pair. However, semantic relations exist not only within each image–recipe pair, but extensively between different pairs. For example, the two image–recipe pairs in Figure 1a belong to the same food (chilli sauce), indicating strong semantic relations (highlighted by red lines) between the two food images as well as the two recipes. The conventional metric learning method (e.g., triplet loss), however, fails to capture this relation information. To be sure, there are lots of image pairs belonging to the same food in practical time. This situation indicates that effectively enhancing intra-modal semantic alignment is significant for improving recipe retrieval performance.

For this purpose, a straightforward method applied in lots of cross-modal retrieval tasks [46,47,48] is to utilize metric learning or contrastive learning strategy within each modality. However, there is a non-trivial issue, i.e., *food image ambiguity*, in cross-modal recipe retrieval that has not been considered. Specifically, foods that look similar may be made from quite different materials and via different preparation methods. Thus, these similar food images correspond to significantly distinct recipes. For example, in Figure 1b, the top food image is a cup of raspberry smoothie, and the bottom one is a bowl of chilli sauce. Theses two foods are visually similar to each other, yet they are crafted from distinct ingredients and have undergone quite different instructions. Unfortunately, existing methods embed their semantics from the two modalities to the common subspace independently. Due to the resemblance in appearance, these two images will be close in the common space, while their corresponding recipes will not. This leads to a dilemma; embeddings that have a large similarity (small distance) in the visual modality may have a small similarity (large distance) in the text modality. As a result, the two modalities are hard to align with each other, and the models are difficult to converge, which reduces the accuracy of retrieval. Stumped by this stand-out drawback, we observe that recipes are the more reliable modality. In other words, foods prepared using similar recipes will have similar visual appearances. Therefore, this study aims to answer the following two questions:

- **Q1:** How can we measure the similarity between ambiguous food images guided by their corresponding recipes?
- **Q2:** How can we further improve the fine-grained semantic alignment between ingredients and instructions within each recipe to support food image similarity measurement?

To this end, we propose a novel cross-modal recipe retrieval method called the **M**ulti-**M**odal **A**lignment Method for **C**ross-**M**odal **R**ecipe Retrieval (**MMACMR**). To answer **Q1**, we design a novel strategy, the **M**ulti-Modal **D**isambiguity and **A**lignment strategy (**MDA** for short), which calculates the intra-modal similarity of recipes and guides the distances between corresponding images. As shown in Figure 1c, the green square is a recipe (chilli sauce) similar to the one shown by the orange square (sweet chilli sauce). Our MDA strategy attempts to pull them close and guide the distance between the green and orange circles (their corresponding images). For **Q2**, considering ingredients play a significant role within instructions, we introduce sentence-level cross-attention to focus on important ingredients in the instructions and further enhance the representations of recipes. In a nutshell, this work is a pioneering effort to further narrow cross-modal heterogeneity between food images and recipes by considering both multi-modal (inter-modal and intra-modal) alignment while mitigating the impact of food image ambiguity.

To sum up, the main contributions of this article are four fold:

- We propose a novel framework called MMACMR which addresses the problem of ambiguous food images in cross-modal recipe retrieval;
- We introduce a novel deep learning strategy named MDA which promotes the alignment of two modalities without adding new parameters;
- We enhance the representation of recipes by focusing on important ingredients within instructions at the sentence level;
- We conduct extensive experiments on the challenging dataset Recipe1M. The results demonstrate that the proposed technique outperforms several state-of-the-art methods.

The remainder of this article is organized as follows. The technical details and specific learning process are outlined in Section 2, the experimental particulars are discussed in Section 3, and we conclude the paper in Section 4.

## 2. Method

In this section, we first present the notations involved in this paper and provide the problem formulation for cross-modal recipe retrieval in Section 2.1. Then, we elaborate on the technique details of our method MMACMR, including the models in Section 2.2, the strategy in Section 2.3, and the algorithm in Section 2.4.

### 2.1. Notations and Problem Formulations

#### 2.1.1. Notations

Without loss of generality, we denote sets as uppercase, handwritten, bold letters (e.g., D) and matrices as uppercase letters (e.g., W). The *i*-th row of W is denoted by $W_{i}$, and the element found in the *j*-th column of *i*-th row in W is denoted as $W_{ij}$. We represent the transpose of a matrix W as $W^{⊤}$. Notation ${∥\cdot ∥}_{2}$ denotes the L2 norm of a matrix. We use softmax(·) to represent the softmax function. To ease reading, we summarize the frequently used notations in Table 1.

#### 2.1.2. Problem Formulations

Let $D={\left\{X_{i}^{v},X_{i}^{r}\right\}}_{i=1}^{n}$ denote a cross-modal recipe dataset comprising *n* image–recipe pairs, where $X_{i}^{v}$ and $X_{i}^{r}=\langle X_{i}^{tit},X_{i}^{ing},X_{i}^{ins}\rangle$ represent the food image and recipe of the *i*-th pair, respectively. $X_{i}^{tit}$, $X_{i}^{ing}$, and $X_{i}^{ins}$ denote the title, list of ingredients, and list of instructions of the recipe, respectively. Note that each title comprises a single sentence, while both ingredients and instructions consist of several sentences. Given a recipe $X_{i}^{r}$ as a query, cross-modal recipe retrieval aims to search for the most similar food image $X_{i}^{v}$ from this dataset D, or vise versa. To enhance consistent feature distribution alignment across food images and recipes, we attempt to optimize an improved recipe encoder $R=f^{r}\left(X^{r};\theta^{r}\right)$ and an image encoder $V=f^{r}\left(X^{v};\theta^{v}\right)$ under the guidance of a novel learning strategy dubbed MDA. This strategy integrates two losses: an *N*-pairs triplet loss $L_{tri}$ to focus on inter-modal semantic alignment and an RGI loss $L_{RGI}$ to focus on semantic consistency within the same modality. By considering both inter-modal and intra-modal alignment, this approach effectively avoids the harmful effects of food image ambiguity. Therefore, the objective function is formulated as follows:

(1) $$\left({\hat{\theta }}^{v},{\hat{\theta }}^{r}\right)=\arg\min_{\theta^{v},\theta^{r}}\left(L_{tri}+\lambda L_{RGI}\right),$$

where $\theta^{v}$ and $\theta^{r}$ are two learnable parameter vectors for image and recipe encoders, and λ is a pre-defined balance parameter.

### 2.2. Framework Overview

**Figure 2:**
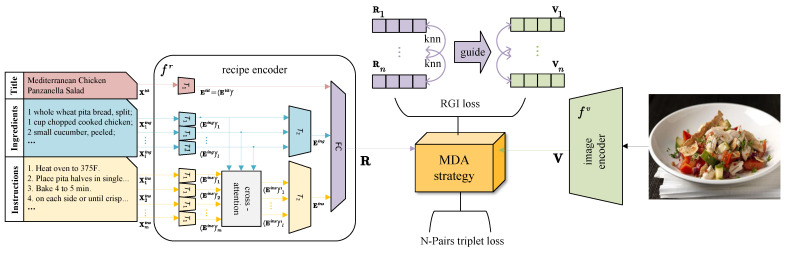
The framework of MMACMR, which comprises two branches of modality encoder, $f^{r}$ for recipe texts and $f^{v}$ for food images, along with the MDA strategy.

An overview of our method MMACMR is depicted in Figure 2. Following prevailing solutions [29,49], the backbone of MMACMR comprises an image encoder $f^{v}\left(\cdot ;\theta^{v}\right)$ and a recipe encoder $f^{r}\left(\cdot ;\theta^{r}\right)$ which project food images and recipes into a common feature subspace. In this subspace, the cross-modal features can be aligned effectively so that the similarity between images and recipes can be measured with accuracy. Below, we provide details of them.

#### 2.2.1. Image Encoder

To fully capture the global semantic relations between fine-grained features in the content of each food image, we adopt the base-size model of Vision Transformer (ViT-B) [50] as the image encoder $f^{v}\left(\cdot ;\theta^{v}\right)$. It is initialized with the weights pre-trained on ImageNet [51] and fine-tuned on the cross-modal recipe dataset. Given a food image $X_{i}^{v}$, the embedding of $X_{i}^{v}$ is denoted as $V_{i}=f^{v}\left(X_{i}^{v};\theta^{v}\right)$.

#### 2.2.2. Improved Recipe Encoder

To focus on the consistent fine-grained semantics between ingredients and instructions, we improve the hierarchical transformer-based recipe encoder [29]. This encoder consists of two levels of transformers, denoted as $T_{1}$ and $T_{2}$, with identical architectures. The first level encodes the title $X_{i}^{tit}$, ingredients $X_{i}^{ing}$, and instructions $X_{i}^{ins}$ at word level and then outputs their sentence-level embeddings, while the second level encoder receives the sentence-level embeddings of ingredients and instructions and produces component-level embeddings. Such a widely adopted recipe embedding scheme unfortunately overlooks a fundamental yet crucial rule in a recipe; the instructions are steps tailored to the ingredients, with the ingredients playing a determining role in shaping the instructions to some extent. To obey this rule, we plug a cross-attention module for instructions between the two transformers for two purposes: (1) to focus on the salient ingredient and (2) to highlight semantic relationships between ingredients and instructions.

Specifically, given a recipe set $\{X_{i}^{r}{\}}_{i=1}^{n}$, as shown in Figure 2, the first level module $T_{1}$ receives the word-level tokens of the three components separately and outputs the average embeddings of every sentence of every components, denoted as $\left({\left(E_{i}^{tit}\right)}^{\prime },{\left(E_{i}^{ing}\right)}^{\prime },{\left(E_{i}^{ins}\right)}^{\prime }\right)=T_{1}\left(X_{i}^{tit},X_{i}^{ing},X_{i}^{ins}\right)$, where ${\left(E_{i}^{ing}\right)}^{\prime }={\left\{{\left(E_{i}^{ing}\right)}_{k}^{\prime }\right\}}_{k=1}^{l}$, ${\left(E_{i}^{ins}\right)}^{\prime }={\left\{{\left(E_{i}^{ins}\right)}_{k}^{\prime }\right\}}_{k=1}^{m}$. To highlight the effect of ingredients to instructions at sentence level and enhance the semantic relationship learning, cross-attention is carried out between ${\left(E_{i}^{ing}\right)}^{\prime }$ and $\left(E_{i}^{ins}\right)$. Firstly, within a recipe, we construct an affinity matrix W as an attention map:

(2) $$W=softmax(\frac{\left({\left(E_{i}^{ing}\right)}^{\prime }W^{ing}\right){\left({\left(E_{i}^{ins}\right)}^{\prime }W^{ins}\right)}^{⊤}}{d}),$$

where ${\left(E_{i}^{ing}\right)}^{\prime }\in R^{l\times d}$, ${\left(E_{i}^{ins}\right)}^{\prime }\in R^{m\times d}$, and *d* is the dimension of each ingredient and each instruction. $W^{ing}\in R^{d\times d}$ and $W^{ins}\in R^{d\times d}$ are learnable weight matrices. Each element $W_{jk}$ means the normalized correlation between the *j*-th ingredient and the *k*-th instruction. Thereby, the embedding of instructions can be enhanced by focusing on the consistent semantics between instructions and ingredients as follows:

(3) $${\left(E_{i}^{ins}\right)}^{\prime \prime }=W{\left(E_{i}^{ins}\right)}^{\prime }.$$

After the second-level processing, we obtain the component-level features of title, ingredients, and instructions: $E_{i}^{tit}$, $E_{i}^{ing}=T_{2}{\left(E_{i}^{ing}\right)}^{\prime }$ and $E_{i}^{ins}=T_{2}{\left(E_{i}^{ins}\right)}^{\prime \prime }$. Finally, these three component embeddings are concatenated and fed into a linear layer; thus, we obtain the final recipe feature, $R_{i}=\mathrm{FC}\left(\left[E_{i}^{tit};E_{i}^{ing};E_{i}^{ins}\right];\theta^{l}\right)$, where $\mathrm{FC}(\cdot ;\theta^{l})$ is a linear layer, $\theta^{l}$ are the parameters of it, and symbol [·;·;·] denotes the concatenation operation.

### 2.3. Multi-Modal Disambiguity and Alignment

To enhance the consistent feature distribution alignment across food images and recipes, we extend the prevailing learning scheme (only inter-modal metric learning, e.g., triplet loss) by considering both inter- and intra-modal alignment. To do so, we employ *N*-pairs triplet loss to realize inter-modal alignment within each batch, while we propose a novel RGI loss to steer the model towards capturing intra-modal consistent semantics effectively by preventing the misrecognition of ambiguous food images.

#### 2.3.1. Inter-Modal Alignment: *N*-Pairs Triplet Loss

Given an anchor food image $V_{i}$, a positive recipe $R_{i}^{+}$, and a negative recipe $R_{j}^{-}$, where i≠j, the *N*-pairs triplet loss for visual modality can be defined as follows:

(4) $$L_{tri}^{v}=\sum_{\left|V\right|}{\left[\left(V_{i},R_{i}^{+},R_{j}^{-}\right)=\left(S\left(V_{i},R_{j}^{-}\right)-S\left(V_{i},R_{i}^{+}\right)+m\right)\right]}_{+},$$

where ${\left[\cdot \right]}_{+}=max\left(0,\cdot \right)$, S(·) is the similarity function (we use cosine similarity here), $\left|V\right|$ is the number of the image sample in the batch, and *m* is a pre-defined margin (we set m=0.3 in this work). Similarly, the *N*-pairs triplet loss for text modality can be written in the same way. Consequently, we formulate the whole *N*-pairs triplet loss as follows:

(5) $$L_{tri}=L_{tri}^{v}+L_{tri}^{r}.$$

#### 2.3.2. Intra-Modal Alignment with Disambiguity: RGI Loss

As discussed above, *N*-pairs triplet loss is a satisfactory scheme for reducing heterogeneity between images and recipes. Using it within each modality, however, is far from a suitable intra-modal alignment solution due to the disturbance of food image ambiguity. Nor is this all; the prevailing recipe retrieval approaches [18,29] only consider cross-modal similarity measurement, which narrows the distance between anchor and positive samples while enlarging the distances between the anchor and negative samples. Such a limitation, on the one hand, leads to a discrepancy between the two modalities, making it difficult for model convergence. On the other hand, it is easy to match one of the ambiguous images, resulting in low retrieval performance.

Fortunately, recipes, or, more rigorously, text, are the more reliable modality owing to their ability to abstract semantic expression word by word. Thus, inspired by [52], we design a novel learning strategy termed RGI loss which chooses the similarity relations between recipes as guidance to determine the relations between corresponding food images. Specifically, if we assume that $<R_{i},R_{j}>$ is a recipe pair in a batch, we aim to preserve the similarity relation for it and project this relation to the corresponding image pair $<V_{i},V_{j}>$. Given a recipe $R_{i}$, we first rank other recipes in this batch by the similarity to $R_{i}$ using the K-nearest neighbors (KNN) algorithm [53]. From the ranked recipes, we select the nearest neighbor as the positive sample $R_{j}^{+}$ and a randomly selected recipe that is not among the top 10 neighbors as the negative recipe $R_{k}^{-}$, i≠j≠k. Inspired by the angular loss [54], our RGI loss for text modality is defined as follows:

(6) $$L_{RGI}^{r}={\left[{∥R_{i}-R_{j}^{+}∥}_{2}^{2}-4\tan^{2}\alpha {∥R_{k}^{-}-C_{i}∥}_{2}^{2}\right]}_{+},$$

(7) $$C_{i}=\frac{R_{i}+R_{j}^{+}}{2},$$

where $\tan^{2}\alpha =1$ is a pre-defined upper bound. For the visual modality, we no longer compute the KNN for images, while we adopt the rank of the neighbors of corresponding recipes directly. The RGI loss for the visual modality is defined in the same way:

(8) $$L_{RGI}^{v}={\left[{∥V_{i}-V_{j}^{+}∥}_{2}^{2}-4\tan^{2}\alpha {∥V_{k}^{-}-C_{i}∥}_{2}^{2}\right]}_{+},$$

(9) $$C_{i}=\frac{V_{i}+V_{j}^{+}}{2},$$

where $\tan^{2}\alpha =1$ is a pre-defined upper bound. Note that the indices of the visual modality are the same as the text modality. Thus, the entire RGI loss is formulated as follows:

(10) $$L_{RGI}=\lambda_{1}L_{GRI}^{r}+\lambda_{2}L_{RGI}^{v},$$

where $\lambda_{1}$ and $\lambda_{2}$ are hyper-parameters for adjusting the relation projection.

#### 2.3.3. Total Loss

Finally, the total loss can be written as follows:

(11) $$L_{total}=L_{tri}+\lambda L_{RGI},$$

where λ is a balance hyper-parameter for adjusting the performance of the two loss functions.

### 2.4. Optimization

Our method undergoes end-to-end optimization. The optimization procedure is outlined in Algorithm 1.
**Algorithm 1** Optimization procedure of MMACMR**Input:** cross-modal recipe dataset $D={\left\{X_{i}^{v},X_{j}^{r}\right\}}_{i,j=1}^{n}$, number of epoch *T*. **Output:** parameters $\theta^{v}$, $\theta^{r}$ of modality encoders.1:Initialize $\theta^{v}$, $\theta^{r}$;2:**for** t=1 to *T* **do**3:   **repeat**4:     Compute embeddings V and R;5:     **for** i=1 to *n* **do**6:        Calculate Equation (5)7:        Rank the recipes neighbors via KNN algorithm;8:        Rank the images neighbors follow recipes;9:        Calculate Equation (10);10:     **end for**11:     Update the parameters $\theta^{v}$, $\theta^{r}$ by Equation (11) via gradient descent algorithm.12:   **until** Convergence13:**end for**

## 3. Experiments and Discussion

This section presents extensive experiments conducted to assess our method’s performance. We begin by introducing the experiment settings, followed by a detailed discussion of the experimental results.

### 3.1. Experiment Settings

#### 3.1.1. Dataset

We implement experiments on the Recipe1M [9] dataset, which is by far the largest public multi-modal recipe dataset available. Recipe1M comprises over 1 million cooking recipe texts and 800 K food images which are collected from more than 24 popular cooking websites. We adhere to the official splits for data, with 238,399 image–recipe pairs allocated for training, 51,119 pairs for validation, and 51,303 pairs for testing.

#### 3.1.2. Baselines

We benchmark our approach against the state-of-the-art baselines below:

- CCA [9] stands for Canonical Correlation Analysis, a classical statistical method used to learn a joint embedding space;
- JE [9] was the first to conduct the cross-modal recipe retrieval task on the Recipe1M dataset. It uses a joint encoder and a classifier to learn the information from food images and recipes;
- AdaMin [10] combines the retrieval loss and classifies the loss to improve the robustness of models and proposes a novel strategy to mine the significant triplets;
- R2GAN [35] promotes the modality alignment by employing a GAN mechanism equipped with two discriminators and one generator;
- MCEN [14] bridges the semantic gap between the two modalities using stochastic latent variable models;
- SN [16] employs three attention mechanisms on three components of recipes to capture the relationship between sentences;
- SCAN [13] introduces semantic consistency loss to regularize the representations of images and recipes;
- HF-ICMA [20] exploits the global and local similarity between the two modalities by considering inter- and intra-modal fusion;
- SEJE [22] constructs a two-phase feature framework and divides the processes of data pre-processing and model training to extract additional semantic information;
- M-SIA [17] argues that multiple aspects in recipes are related to multiple regions in food images and leverages multi-head attention to bridge them;
- X-MRS [39] augments recipe representations by utilizing multilingual translation;
- LCWF-GI [31] employs latent weight factors to fuse the three components of recipes by considering their complex interaction;
- H-T [29] captures the latent semantic information in recipes by applying self-supervised loss to push components sourced from the same close recipe;
- LMF-CSF [30] introduces a low-rank fusion strategy to combine the components in recipes and generate superior representations.

#### 3.1.3. Evaluation Criteria

Similar to the majority of previous studies [9,29,44], we sample 1 K and 10 K image–recipe pairs from the test partition and assess the retrieval performance for image-to-recipe and and recipe-to-image tasks using median rank (MedR) and recall rate at top *k* (R@*k*). Among these metrics, MedR represents the median index of the retrieved samples for each query, measuring the ability of models to understand the semantic correlation between two modalities and the accuracy of retrieval. A lower MedR value indicates better performance. R@*k* indicates that the percentage of the ground truth index is among the first *k* retrieved samples, which is also known as sensitivity or the true positive rate, measuring the ability of models to correctly identify all relevant instances. A higher R@*k* value indicates better performance. Here, we evaluate the top 1 (R@1), top 5 (R@5), and top 10 (R@10). By using these two metrics, we can evaluate the comprehensive performance of the models. Every evaluation is repeated 10 times, and the mean results are returned.

#### 3.1.4. Implementation Details

In line with prior research [49], we use food images with a depth of three channels in the RGB color space. All the images in our experiments are resized to 256 pixels in their shorter dimension and then cropped to 224×224 pixels. The image encoder utilizes a pre-trained ViT-based model, yielding an output size of 1024. Regarding recipes, sentences in three components are truncated to a maximum length of 15, and every ingredients or instructions list has a maximum of 20 sentences. Each transformer in the hierarchical transformer recipe encoder comprises two layers, and each layer has four attention heads. Every component in the recipes is embedded as 512 dimensions, and the final embedding of a recipe is output as 1024 dimensions. The model is trained utilizing the Adam optimizer, the batch size is set as 128, and the learning rate is $\eta =10^{-4}$. The balance parameters $\lambda_{1}=0.09$, $\lambda_{2}=0.1$, and λ=0.01.

#### 3.1.5. Experimental Environment

Our experiments are conducted using Python 3.7 with the PyTorch 1.31.1 framework. We utilize a deep learning workstation equipped with an Intel(R) Core i9-12900K 3.9 GHz processor, 128 GB of RAM, 1 TB SSD, and 2 TB HDD storage. The workstation runs on the Ubuntu-22.04.1 operating system and is powered by two NVIDIA GeForce RTX 3090Ti GPUs (NVIDIA, Palo Alto, CA, USA).

### 3.2. Comparison with State-of-the-Art Methods

We compare the performance of our method with the baselines mentioned above. The results are reported in Table 2. It is easy to see that MMACMR is superior to the best results of existing works using all the metrics. Concretely, our method achieves a 3.3, 1.1, 0.6 R{1, 5, 10} improvement for image to recipe and a 3.7, 1.2, 0.7 R{1, 5, 10} improvement for recipe to image in the 1 K size compared to the SOTA method LMF-CSF [30] and achieves a 3.5, 3.1, 2.7 R{1, 5, 10} improvement for image to recipe and a 4.0, 3.1, 2.8 R{1, 5, 10} improvement for recipe to image in the 10 K size compared to the SOTA method LMF-CSF [30]. In addition, the MedR of our method in the 10 K size dataset decreases to 2.1 for image to recipe and 2.2 for recipe to image compared to 3.0 in LMF-CSF [30]. These results demonstrate the effectiveness of our MMACMR. In other words, our approach to addressing the questions mentioned above is effective for cross-modal recipe retrieval.

### 3.3. Scalability Analysis

**Figure 3:**
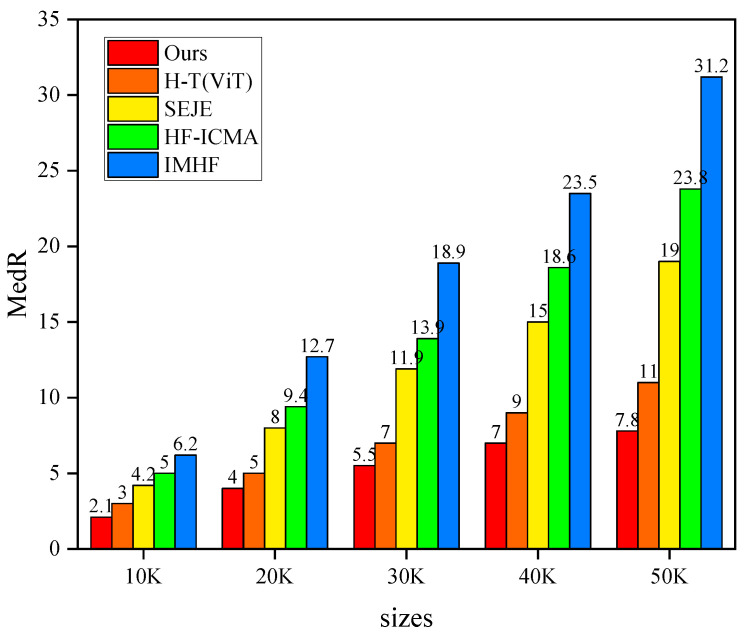
Scalability analysis. The abscissa represents the dataset size ranging from 10 K to 50 K, while the ordinate represents the MedR value.

In order to investigate the scalability of our method, we conduct experiments on datasets larger than 10 K in size. As shown in Figure 3, the MedR results of MMACMR are consistently lower than those of all other methods across all dataset sizes. In addition, it can be seen that, with the increase in test size, the performance gap between our method and others also widens. We argue that, on the one hand, the enhancement of recipe embedding promotes the alignment between the two modalities. On the other hand, as the dataset size increases, so does the number of ambiguous food images, leading to a higher probability of matching incorrect recipes. By effectively addressing this issue, our method demonstrates improved robustness and scalability as the dataset size enlarges.

### 3.4. Ablation Studies

In this subsection, we conduct ablation experiments to assess the contribution of each part of our model to the overall performance. Table 3 reports the image-to-recipe retrieval results of different parts of MMACMR in 1 K and 10 K test size. In Table 3, Base is the baseline framework consisting of the food image encoder (ViT-B) and the original hierarchical transformer recipe encoder coupled with the *N*-pairs triplet loss. IR means introducation of the improved recipe encoder, and $L_{RGI}$ is our RGI loss. A √ symbol under the columns Base, IR, and $L_{RGI}$ indicates the use of that part. On the right, we list the MedR, R@1, R@5, and R@10 results for the image-to-recipe and recipe-to image tasks. We first evaluate the Base framework, then introduce the improved recipe encoder and RGI loss separately. Finally, we combine all three parts. It can be observed that the addition of both IR and $L_{RGI}$ boosts the baseline model. This indicates that the solutions we propose to address the questions mentioned above are effective. When employing all subassemblies, we achieve the best performance, further validating the effectiveness of each element in our approach. Note that the method without IR obtains the same scores as the full method in R@5 and R@10 for image to recipe, and R@5 for recipe to image, for the 10 K size dataset. Additionally, it achieves better performance in MedR for recipe to image in 10 K size. Therefore, we attribute the main contribution to the MDA strategy.

### 3.5. Qualitative Results

#### 3.5.1. Qualitative Results on Image-to-Recipe Retrieval

**Figure 4:**
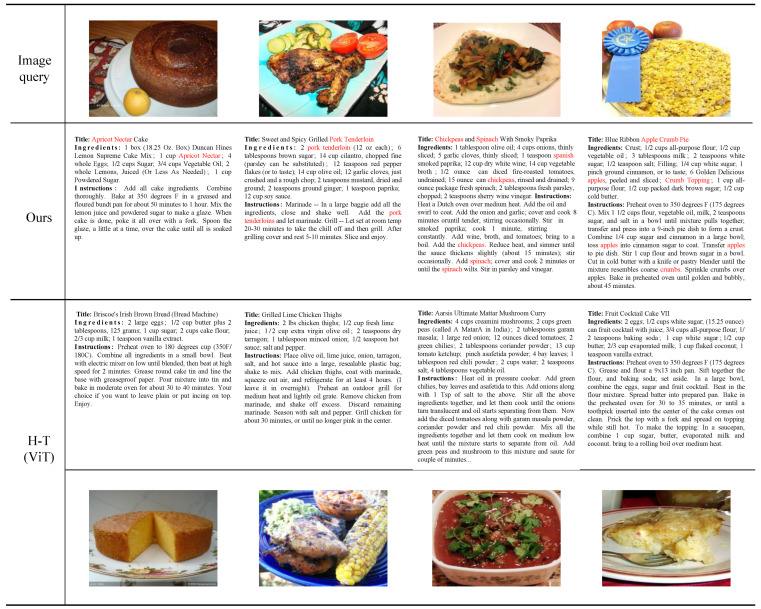
Examples of image-to-recipe retrieval results for the 10 K test set. The first row contains the query images, the second row shows the recipes retrieved using our method (all of which are the ground truth recipes; therefore, the first row is their corresponding food images), the third row displays the recipes retrieved using H-T (ViT) [29], and the last row presents the corresponding food images of the recipes from the third row. The key ingredients not retrieved by H-T [29] but retrieved by our method are highlighted in red.

To more intuitively analyze the representative results of MMACMR in image-to-recipe retrieval, we select four food images as queries to retrieve the recipes from the test set using our method and the SOTA method H-T (ViT) [29]. As shown in Figure 4, from left to right, the queries are “Chickpeas and Spinach with Smoky Paprika”, “Blue Ribbon Apple Crumb Pie”, “Apricot Nectar Cake”, and “Sweet and Spicy Grilled Pork Tenderloin”. In the first two samples, the categories of food are relatively easy to distinguish; both of these methods retrieve approximate recipes. However, in the first example, H-T (ViT) [29] does not retrieve the main ingredient, apricot nectar, while our method successfully retrieves it. The same situation occurs in the second example, where H-T (ViT) [29] retrieves a recipe whose corresponding image is similar to the query image but it misrecognizes the pork tenderloin as chicken thighs. In contrast, MMACMR retrieves the ground truth recipe. We attribute this to our MDA strategy, which can better address the problem of ambiguous food images and recognize the ingredients correctly. In the third example, H-T (ViT) [29] identifies some beans and vegetable leaf in the image but misclassifies their types, and the retrieved entire recipe deviates significantly from the ground truth. In the last example, the food image is difficult to recognize by human eye. H-T (ViT) [29] retrieves a recipe whose corresponding image has a similar color to the query (actually, it is a shortcut for models to classify objects which have not been seen before). However, our method retrieves the correct recipe even though the query image is ambiguous. We believe this is because MMACMR can reduce the distances between images with similar recipes, allowing the correct sample to be retrieved even when the query is hard to distinguish.

#### 3.5.2. Qualitative Results on Recipe-to-Image Retrieval

**Figure 5:**
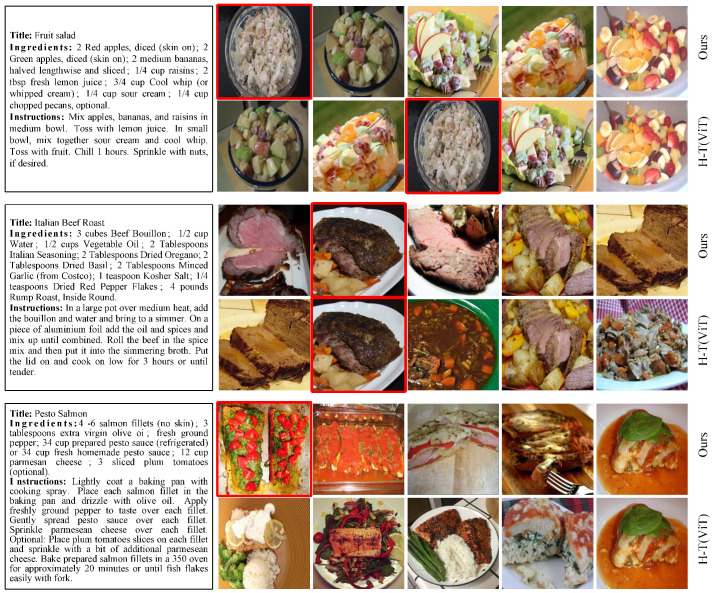
Examples of recipe-to-image retrieval results for the 10 K test set. The left side shows the query recipes, while the right side displays the top 5 retrieved images using our method or H-T (ViT) [29]. The ground truth images are marked by a red box.

We also conduct experiments to visualize the results of recipe-to-image retrieval for the 1 K test set, which are presented in Figure 5. From top to bottom, the query recipes are titled “Fruit Salad”, “Italian Beef Roast”, and “Pesto Salmon”, followed by the top five retrieved images using our method and the SOTA method H-T (ViT) [29]. In the first example, both methods retrieve five food images of fruit salad, but our method retrieves the ground truth as the top one, while H-T (ViT) [29] retrieves it in the top three. In the second example, the two methods retrieve the correct image in the top two. However, all the food images MMACMR retrieves are roast beef, while the third and fifth retrieved images of H-T (ViT) [29] do not match the recipe query. In the last example, our method retrieves the ground truth image as the top one, while H-T (ViT) [29] fails to retrieve the correct food image. At the same time, the second image retrieved by MMACMR is similar to the correct one, while the first and third images retrieved by H-T (ViT) [29] deviate significantly from the ground truth. We attribute these achievements to the capability of our method to address the problem of ambiguous food images, allowing MMACMR to retrieve images that have similar recipes.

## 4. Conclusions

In this paper, we propose a novel cross-modal recipe retrieval method named MMACMR which addresses the problem of ambiguous food images in retrieval using a novel training strategy, MDA, that guides the similarity within food images by recipe. Additionally, we improve the recipe encoder to ensure the precision of recipe embeddings. We conduct extensive experiments on the challenging public dataset Recipe1M, and the experimental results demonstrate the effectiveness of our method. Given the necessity of analyzing vast numbers of food data, our method could offer significant practical value in the food industry by enhancing user convenience and efficiency.

However, due to the complexity of recipe texts, some information representing the dish preparation program is still not captured by our method. In the future, we aim to focus on the fine-grained information in recipes using visual language pre-training models.

## Figures and Tables

**Table 1:** Summary of notations.

Notation	Definition
D	A cross-modal recipe dataset
$X_{i}^{v}$	The food image of the *i*-th pair
$X_{i}^{r}$	The recipe of the *i*-th pair
$X_{i}^{tit}$	The title of the recipe $X_{i}^{r}$
$X_{i}^{ing}$	The ingredients of the recipe $X_{i}^{r}$
$X_{i}^{ins}$	The instructions of the recipe $X_{i}^{r}$
$E_{i}^{tit}$	The embedding of the title in a recipe $X_{i}^{r}$
$E_{i}^{ing}$	The embedding of the ingredients in a recipe $X_{i}^{r}$
$E_{i}^{ins}$	The embedding of the instructions in a recipe $X_{i}^{r}$
R	The recipe embedding
V	The food image embedding
$f^{r}$	The recipe encoder
$f^{v}$	The image encoder
$\theta^{r}$	The parameters of recipe encoder
$\theta^{v}$	The parameters of image encoder
$L_{tri}$	The *N*-pairs triplet loss function
$L_{RGI}$	The RGI loss function

**Table 2:** Comparison with SOTA methods. MedR(↓) and R@k(↑) in 1 K and 10 K size. The best results are marked in bold font.

	Methods	Image to Recipe				Recipe to Image			
		MedR	R@1	R@5	R@10	MedR	R@1	R@5	R@10
1 K	CCA [9]	15.7	14.0	32.0	43.0	24.8	9.0	24.0	35.0
	JE [9]	5.2	24.0	51.0	65.0	5.1	25.0	52.0	65.0
	AdaMin [10]	2.0	39.8	69.0	77.4	2.0	40.2	68.1	78.7
	R2GAN [35]	2.0	39.1	71.0	81.7	2.0	40.6	72.6	83.3
	MCEN [14]	2.0	48.2	75.8	83.6	1.9	48.4	76.1	83.7
	ACME [34]	1.0	51.8	80.2	87.5	1.0	52.8	80.2	87.6
	SN [16]	1.0	52.7	81.7	88.9	1.0	54.1	81.8	88.9
	SCAN [13]	1.0	54.0	81.7	88.8	1.0	54.9	81.9	89.0
	HF-ICMA [20]	1.0	55.1	86.7	92.4	1.0	56.8	87.5	93.0
	SEJE [22]	1.0	58.1	85.8	92.2	1.0	58.5	86.2	92.3
	M-SIA [17]	1.0	59.3	86.3	92.6	1.0	59.8	86.7	92.8
	X-MRS [39]	1.0	64.0	88.3	92.6	1.0	63.9	87.6	92.6
	H-T [29]	1.0	60.0	87.6	92.9	1.0	60.3	87.6	93.2
	LCWF-GI [31]	1.0	59.4	86.8	92.5	1.0	60.1	86.7	92.7
	H-T(ViT) [29]	1.0	64.2	89.1	93.4	1.0	64.5	89.3	93.8
	LMF-CSF [30]	1.0	65.8	89.7	94.3	1.0	65.5	89.4	94.3
	Ours	**1.0**	**69.1**	**90.8**	**94.9**	**1.0**	**69.2**	**90.6**	**95.0**
10 K	JE [9]	41.9	-	-	-	39.2	-	-	-
	AdaMin [10]	13.2	14.9	35.3	45.2	12.2	14.8	34.6	46.1
	R2GAN [35]	13.9	13.5	33.5	44.9	12.6	14.2	35.0	46.8
	MCEN [14]	7.2	20.3	43.3	54.4	6.6	21.4	44.3	55.2
	ACME [34]	6.7	22.9	46.8	57.9	6.0	24.4	47.9	59.0
	SN [16]	7.0	22.1	45.9	56.9	7.0	23.4	47.3	57.9
	SCAN [13]	5.9	23.7	49.3	60.6	5.1	25.3	50.6	61.6
	HF-ICMA [20]	5.0	24.0	51.6	65.4	4.2	25.6	54.8	67.3
	SEJE [22]	4.2	26.9	54.0	65.6	4.0	27.2	54.4	66.1
	M-SIA [17]	4.0	29.2	55.0	66.2	4.0	30.3	55.6	66.5
	X-MRS [39]	3.0	32.9	60.6	71.2	3.0	33.0	60.4	70.7
	H-T [29]	4.0	27.9	56.4	68.1	4.0	28.3	56.5	68.1
	LCWF-GI [31]	4.0	27.9	56.0	67.8	4.0	28.6	55.8	67.5
	H-T(ViT) [29]	3.0	33.5	62.1	72.8	3.0	33.7	62.2	72.7
	LMF-CSF [30]	3.0	34.6	62.7	73.2	3.0	34.3	62.5	72.8
	Ours	**2.1**	**38.1**	**65.8**	**75.9**	**2.2**	**38.3**	**65.6**	**75.6**

**Table 3:** Ablation study. MedR (↓) and R@k (↑) in 1 K and 10 K size. The best results are marked in bold font. A √ symbol indicates that the corresponding part in this column is being used.

	Base	I R	$L_{\mathrm{RGI}}$	Image to Recipe				Recipe to Image			
				MedR	R@1	R@5	R@10	MedR	R@1	R@5	R@10
1 K	√			1.0	58.3	86.2	91.8	1.0	59.6	86.1	92.2
	√	√		1.0	67.2	90.0	94.5	1.0	67.6	90.0	94.5
	√		√	1.0	68.6	90.5	94.7	1.0	68.2	90.3	94.7
	√	√	√	**1.0**	**69.1**	**90.8**	**94.9**	**1.0**	**69.2**	**90.6**	**95.0**
10 K	√			4.1	26.8	54.7	66.5	4.0	37.5	55.1	66.8
	√	√		3.0	35.9	64.5	74.7	3.0	36.6	64.7	74.9
	√		√	2.2	37.7	65.8	75.9	**2.0**	38.0	65.6	75.4
	√	√	√	**2.1**	**38.1**	**65.8**	**75.9**	2.2	**38.3**	**65.6**	**75.6**

## Data Availability

The original contributions presented in the study are included in the article, further inquiries can be directed to the corresponding author.

## Abbreviations

The following abbreviations are used in this manuscript:

MMACMR	Multi-Modal Alignment Method for Cross-Modal Recipe Retrieval
MDA	Multi-Modal Disambiguity and Alignment
RGI	Recipe Guide Image
AI	Artificial intelligence
LSTM	Long Short-Term Memory
GANs	Generative Adversarial Networks
ViT	Vision Transformer
CLIP	Contrastive Language–Image Pre-training
KNN	K-Nearest Neighbors
SOTA	State Of The Art
MedR	Median Rank
SSD	Solid-State Disk
RAM	Random-Access Memory
HDD	Hard Disk Drive
CCA	Canonical Correlation Analysis
